# Early evidence of sheep lambing de-seasoning in the Western Mediterranean in the sixth millennium BCE

**DOI:** 10.1038/s41598-020-69576-w

**Published:** 2020-07-30

**Authors:** Carlos Tornero, Marie Balasse, Stéphanie Bréhard, Isabelle Carrère, Denis Fiorillo, Jean Guilaine, Jean-Denis Vigne, Claire Manen

**Affiliations:** 1 Archéozoologie, archéobotanique: sociétés, Pratiques Et Environnements (AASPE), Muséum National D’histoire Naturelle, CNRS, CP 56, 55 rue Buffon, 75005 Paris, France; 2 Laboratoire TRACES - UMR 5608, Université Toulouse - Jean Jaurès Maison de La Recherche, 5, allée Antonio Machado, 31058 Toulouse (Cedex 9), France; 3 Collège de France, 11, Place Marcelin-Berthelot, 75005 Paris, France; 4 Present Address: Institute of Human Paleoecology and Social Evolution (IPHES), Zona Educacional 4, Campus Sescelades URV (Edifici W3), 43007 Tarragona, Spain

**Keywords:** Archaeology, Archaeology

## Abstract

Today, sheep farmers in the Western Mediterranean de-season their ewes to achieve autumnal births. This strategy contrasts sharply with spring lambing further north, and provides benefits in terms of out-of-season availability of animal products. These competences are closely linked to specific sheep physiology and favorable Western Mediterranean climatic conditions. It is not known exactly how far back in the past the ability to de-season Mediterranean sheep breeds extends. This study shows that this practice existed seven millennia ago in Southern France, at an early stage of the major agricultural expansion of the Neolithic into the Western Mediterranean. To achieve this reproductive management regime, three prerequisites were required: (i) the ability of sheep to give birth in autumn, constituting early evidence for the genetic selection of specimens with prolonged reproductive fertility; (ii) intentional management of female and male interactions within the herd, which would have required good knowledge of the timing of the fertility cycle in ewes, and; (iii) adequate pasture resources to support lactation in the autumn, possibly favored by autumnal rains, substantiating previous paleoclimatological data for the existence of a Mediterranean-type precipitation regime at that time. Moreover, we also show that winter foddering of sheep occurred, using forest resources, and that this maintained good body weights for spring mating. These findings add pivotal information about shepherding practices and the socio-economic abilities of Early Neolithic communities, which allowed for the extension of their areas of influence from the Eastern Mediterranean area to the West during the Early Neolithic agricultural expansion in Europe.

## Introduction

After the initial domestication of sheep in Southeast Anatolia c. 8,500 cal BCE^1^^,^ domestic lineages (*Ovis aries*, L. 1758) were introduced into Europe as part of the wave of agricultural population dispersal during the Early Neolithic towards the west and north of Europe, between 6,800 and 3,500 cal BCE^2,3^. In the Western Mediterranean, domestic sheep were first introduced by pioneering groups from the *Impressa* culture from the beginning of the sixth millennium BCE to Southern Italy and shortly afterwards in the South of France. This first diffusion wave was followed by a second wave of human groups from the Cardial/Epicardial cultural complex, between 5,450 and 4,700 cal BCE in the South of France and in north-eastern Spain, marking the full development of Neolithic agropastoral economies in these areas^4–6^. During the eight millennia of history separating present-day farming from the earliest implementations in the Western Mediterranean, the cultural and biological components of husbandry systems were reshaped. This included modifications in sheep genetic heritage through selection and population redistributions and changes in practices (i.e. herding, management of diet and age and sex composition of the herds), due to mutations in the social sphere, as well as in economic rules^7^.

Today, a strong identity of farming practices in the Mediterranean margins of France and Spain is defined by sheep husbandry systems with autumnal lambing as the main lambing season^8–10^, although a certain variability exists in the lambing season according to the characteristics of the herds, the type of production and the practice or not of transhumance. Most importantly, in this area, the autumnal lambing is practiced without artificial manipulation involving light or hormonal treatments. This contrasts significantly with husbandry systems implemented further north in Europe, where sheep give birth from late winter and throughout the spring^11^^,^ and where the same timing was also described as predominant during the Neolithic period under latitudes ∼ 43° to 59°N^12^. The timing of domestic stock reproduction is a key parameter in husbandry economies in general. Sheep, in particular, have seasonal reproductive behavior in temperate latitudes, imposing strong constraints on work organization and the availability of animal products^13^. Autumnal lambing, designated today as “out-of-season” lambing, is prized, mainly because it brings benefits in terms of out-of-season availability of sheep products.

In the Western Mediterranean area, autumnal lambing is made possible by a dual specificity in both sheep physiology and the environment. Sheep have inherited from their wild ancestor a seasonal reproductive strategy. Under temperate latitudes, the annual alternation of the cycle of fertility and infertility periods in ewes is under strong photoperiodic control^14,15^, defining a period in the year when most ewes in a herd are not sexually active. The sheep breeding season typically occurs from early autumn to later winter. In contrast, in some sheep breeds originating and living in Mediterranean latitudes, the breeding season starts in late summer and can be prolonged until mid-spring^8,16–18^. In practical terms, a prolonged fertility period enables farmers to schedule mating in the spring, leading to autumnal births after a five-month gestation period. Moreover, in the Western Mediterranean, autumnal lambing is also favored by environmental factors: the autumnal rains and renewed vegetation create conditions that support autumnal lactation. Typically, in south-eastern France, the conjunction of both of these favorable factors forms the basis for Mérinos d’Arles breed husbandry systems, involving transhumance to the Alpine mountains in summer and two lambing seasons. Sheep are preferentially mated in the spring, enabling them to give birth in the autumn when returning from summer pastures,while a “catch-up” lambing season is scheduled for the spring^9^.

This increased ability for de-seasoning Mediterranean sheep breeds is not the result of recent manipulation. It is generally considered to be related to a regionally specific history^10^^,^ although it is unclear exactly how far back in the past this practice extends. The history of de-seasoning sheep cannot be distinguished from the history of the Mediterranean climate in the Western Mediterranean region. Recent paleoclimatic studies have indicated the importance of seasonal precipitation patterns in defining the past Mediterranean climate^19,20^. Although most authors previously accepted a relatively recent date of around 2,500 cal BCE^21,22^ for the establishment of the present-day Mediterranean climate, pollen-based quantitative estimates of seasonal precipitation from the Central Mediterranean (Lake Accesa, central Italy) have highlighted the existence of a seasonal Mediterranean precipitation pattern, with summer droughts and maximum rains in the autumn/winter during the Holocene optimum (7,500–5,800 cal BCE)^19^. This optimum period was followed by a phase of gradual aridification, with a progressive decline in winter precipitation, culminating around 3,000 cal BCE^19^. The first agropastoral societies spread into the Western Mediterranean region immediately prior to and during the first stages of this gradual aridification phase, and greater climatic variability ensued for several centuries, but was still characterized by similar or higher winter precipitation than present-day levels^19^. Consequently, it can be assumed that favorable climatic and seasonal vegetal dynamics might have existed, which could have enabled autumnal lambing and lactation in the Early Neolithic in the Western Mediterranean.

Apart from the necessity of providing adequate grazing resources at lactation time for the survival of the lambs, autumnal lambing would also have required careful management of sheep nutrition to enhance fertility at the mating time. The nutritional state can be a powerful regulator of sheep reproduction^23,24^. In particular, maintaining good body weight throughout the winter for spring mating is critical in a system promoting autumnal lambing. Therefore, it is also reasonable to ask how far back such specific foddering practices can be attested and what resources may have been used for this purpose. The use of arboreal fodder (leaves and twigs) to overwinter livestock has been documented in traditional husbandry systems in the Mediterranean area^25^. It is demonstrated across most of Europe until a few centuries ago and was directly confirmed based on archaeobotanical evidence in numerous Early and Middle Neolithic contexts^26–28^. Forested landscapes prevailed in the Early Neolithic in south-eastern France, where farming activities did not lead to significant forest clearance until the Middle Neolithic, i.e., from the fourth millennium BCE onwards^29,30^. It can therefore be hypothesized that forests potentially provided resources to complement the sheep diet during winter.

The physiological aptitude of Early Neolithic sheep in the Western Mediterranean for autumnal lambing is still an unknown parameter in these husbandry systems. The present study investigates how early sheep husbandry systems with autumnal lambing may have been in place in the Western Mediterranean, focusing on two sites from the Early Neolithic Cardial/Epicardial complex in Southern France: Taï (Remoulins) and Gazel (Sallèles-Cabardès)^31,32^. Both sites contained sheep tooth remains which were used to investigate lambing and food management on a seasonal scale using sequential measurements of stable oxygen (δ^18^O) and carbon (δ^13^C) isotope ratios in enamel. Indeed, the results show that the first agropastoral systems in Southern France met the biological and environmental conditions for autumnal lambing and that the contribution of forest resources in winter could have favored spring mating by maintaining ewes in good condition throughout the winter.

### Site selection and interpretative framework

**Figure 1:**
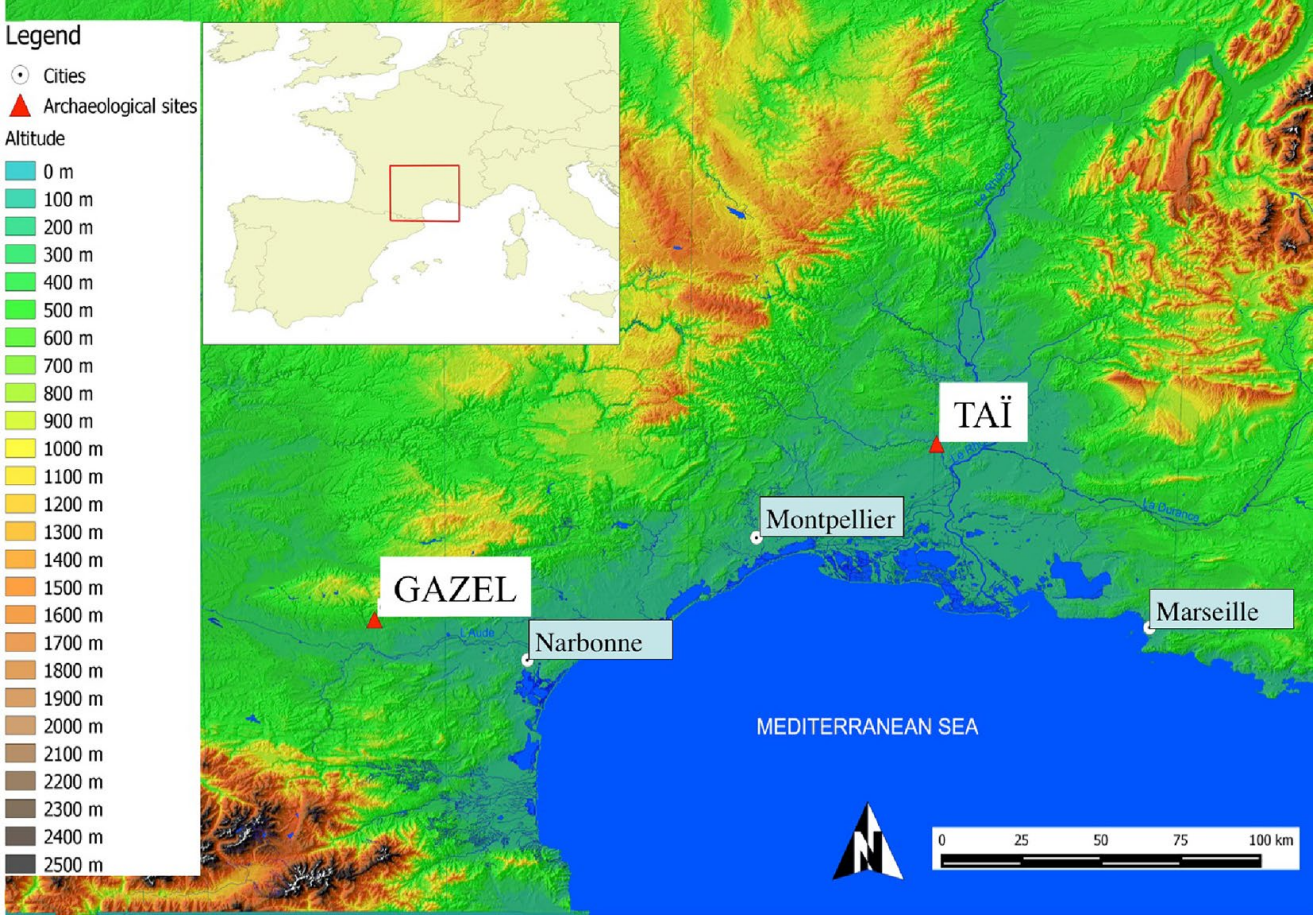
Map showing the location of the Taï and Gazel sites. Map prepared using QGis 2.4.Chugiak software (QGIS Geographic Information System, “Open Source Geospatial Foundation Project” https://www.qgis.org/es/site/). The GeoTiff data used belong from the Institut National de l'Information Geographique et Forestière (http://www.ign.fr). The hydrology data (shapfile format) was obtained from Natural Earth (free vector and raster map data, in ) later modified in Inkscape (https://www.inkscape.org/es).

The two archaeological sites selected for this study are the Gazel and Taï settlements, both located in Southern France in the Languedoc region (∼43° latitude) (Figure 1; Table S1). Gazel (Sallèles-Cabardès, 250 m.a.s.l.) is located in the foothills of a mountainous region at the south-western end of the Massif Central. Taï (Remoulins, 54 m.a.s.l.) is located in a valley opening onto the plain of Remoulins, close to the Rhône Valley. Both sites have been the focus of interdisciplinary archaeological research projects for the last 40 years^33^^,^ and have provided key information for understanding the early Neolithization process in the Western Mediterranean region. They supplied important data on *Cardial* and *Epicardial* occupations, a well-attested period in other contemporaneous sites, such as Montclus (Montclus, Gard), Camprafaud (Ferrières-Poussarou), Oullins (Le Garn, Gard), Corrège (Leucate, Aude) and Mas de Vignoles VI-X (Remoulins, Gard)^34^^,^^4^. More detailed information about the Taï and Gazel sites is presented in SI Materials and Methods.

The analysis focused on the *Epicardial* archaeological levels at both sites. At the Taï site, the *Epicardial* phase is dated to 5,270–4,990 cal BCE in levels C1 and C2^5^^,^ while at the Gazel site, it is found in Phases II and III, dated to 5,350 to 5,200 cal BCE and 5,200 to 5,050 cal BCE (Guilaine and Manen, in progress), respectively. Both sites were dwelling sites, and were occupied throughout the year. At Taï, the presence of storage pits, evidence for agriculture close to the site, and the nature of the archaeobotanical assemblage suggest a permanent occupation and the presence of domestic animals at the site at least most of the year^35,36^. Gazel seems to have been frequented repeatedly throughout the Early Neolithic (phases I to IV, the latter dated around 4,700 BCE), with phases of permanence more pronounced than others. The functional status of the cave is uncertain. Although its occupations might have been related to pastoralism, it may not be defined as a sheepfold-cave. At Taï, the C1 and C2 levels delivered a faunal spectrum predominated by domestic sheep, goats and cattle (91% of the NISP excluding lagomorph remains), where sheep are clearly the main domestic species (71% of the NISP) (Bréhard et al., in progress and SI). At Gazel, the faunal spectra in Phases II and III were largely dominated by domestic caprines (circa 60% of the NISP)^37,38^. At both sites, the mortality profile for domestic caprines is indicative of non-specialized management strategies, suggesting that both meat and milk could have been exploited (Bréhard et al., in press).

In total, 28 teeth belonging to nine sheep from the Taï site and 12 sheep from the Gazel site were selected for stable isotope analysis (*Material and methods and SI*). The sequential isotopic analysis of sheep tooth enamel allows for a reconstruction of individual isotopic histories over the period of tooth formation^39^. Oxygen and carbon derived from ingested plants and water are incorporated in the enamel mineral fraction (bioapatite) during tooth enamel formation^40,41^. Their stable isotopic compositions (δ^18^O and δ^13^C) are measured conjointly in bioapatite carbonate.

The δ^18^O values in tooth enamel are mainly derived from ingested water. In temperate latitudes, δ^18^O values in the skeletons of large herbivores are strongly correlated to the values of local meteoric water^40,42–44^ available to herbivores from surface water and plant water. At high and middle latitudes, the δ^18^O composition of precipitation varies with ambient temperature, with the highest values occurring during the warmest months and the lowest during the coldest months^45–47^. Once retrieved from the dental enamel, these seasonal trends display cyclical variation, which can be used to reconstruct seasonal birth patterns. As the timing of tooth eruption and growth is fixed within a species, inter-individual variability in the δ^18^O sequences retrieved from the enamel is related to variability in the birth season^48–50^. In order to reconstruct birth seasonality, inter-individual variability is described through the observation of the position of the highest δ^18^O value in the tooth crown. A quantitative estimation of variability involves a normalization procedure through modeling^51^ (see *Material and methods*). The lambing season of the archaeological specimens can then be determined by comparison with modern reference sets with known dates of birth^12,52,53^. This approach has been applied successfully to sheep second (M2) and third molars (M3) in previous studies^12,53^.

The δ^13^C values in tooth enamel bioapatite are derived from ingested plants^41,54^. Carbon stable isotope ratios in plants vary according to the photosynthetic pathway and prevailing growing conditions^55,56^. In the Early Neolithic, C_3_ plants dominated vegetal landscape composition in Southern France and no cultivated C_4_ plants may be expected at that time^36,57^ . A diet of C_3_ plants from open areas should produce δ^13^C values around − 11.8‰ in archaeological sheep enamel (*Material and methods*), ± 1‰ to account for seasonal variations^58^. If sheep were foddered, which implies the use of forest-tree leaves, this would lead to lower δ^13^C values due to the canopy effect, where the reduction of light intensity causes a depletion of ^13^C in plants^59,60^. In Southern France, the Late Mesolithic mature oak-dominated forests persisted during the Early Neolithic with only very moderate clearances^29^. Still, the magnitude of the canopy effect on plant δ^13^C values differs according to forest type and is therefore difficult to predict. ^13^C-depletions of 2–5‰ in comparison with plants in neighboring open settings have been reported^58,60^. In archaeological sheep enamel, we estimate that winter δ^13^C values significantly different to − 12.8‰ and closer to − 14.5‰ could reflect a contribution of plant resources gathered in the forest (*Material and Methods*).

## Results

Intra-tooth sequences of δ^18^O and δ^13^C values are shown in Figures S1 to S3. The results are summarized in Table S2.

### Lambing season

**Figure 2:**
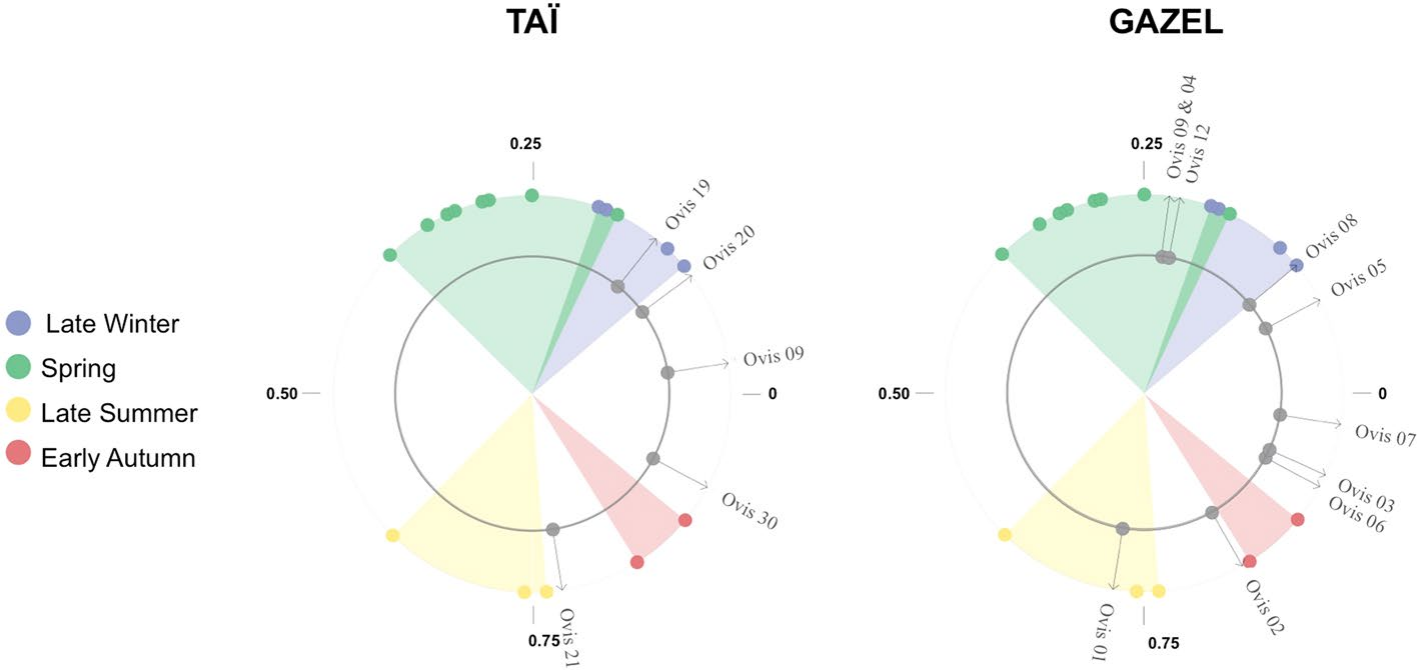
Distribution of sheep births at the Taï and Gazel Early Neolithic sites, as reflected by the position of the maximum δ^18^O value in tooth crown (x_0_) normalized to the period of the cycle (X). The birth season is compared with modern reference sheep (Carmejane CAR: Blaise and Balasse, 2011; Rousay ROU: Balasse et al., 2012a, 2017; Selgua XT: Tornero et al., 2018). Blue, green, yellow and red color areas represent normalized range values obtained from modern specimens (colored dots). Archaeological sheep are represented in grey dots. Detailed information about modern sheep specimens is presented in SI Sheep modern reference sets.

In all the sampled teeth, a pattern of sinusoidal variation in δ^18^O values reflects the seasonal cycle. Birth distribution and lambing seasons were evaluated from the δ^18^O sequences observed in the M2 molar, for which modern reference sets for lambing seasons are available. The modeling procedure was applied to all M2s where a minimum and maximum δ^18^O value could be identified (this excludes specimens Ovis 11 and Ovis 18 at Taï, and specimens Ovis 10 and Ovis 11 at Gazel). The modeling results are shown in Table S3. Inter-individual variability in the position of the maximum δ^18^O value in the tooth crown (x_0_/X), reflecting variability in the birth date, is presented in Fig. 2. These results were then compared to modern reference data sets from sheep specimens with a known birth season (see SI Materials and Method). At both sites, the results suggest seasonal lambing, occurring partly in the autumn (Ovis 21 and 30 from Taï and Ovis 01, 02, 03, 06 and 07 from Gazel), and partly in late winter until spring (Ovis 09, 19 and 20 from Taï and Ovis 04, 05, 08, 09 and 12 from Gazel).

### Seasonal dietary habits

The δ^13^C values measured in sheep teeth from the Taï and Gazel sites suggest a diet based on C_3_ plants throughout the year. The changes in δ^13^C values along the tooth crown appear to be in keeping with the variation observed in the δ^18^O values, reflecting changes in the sheep diet in tune with the seasonal climatic cycle. However, during the winter period (when the lowest δ^18^O values are recorded), the δ^13^C values are lowered to a level exceeding the expected seasonal variation in pasture C_3_ plants (− 27 ± 1‰^58^ or − 11.8 ± 1‰ in enamel). At Taï, in 11 of the 14 analyzed teeth, winter δ^13^C values drop below − 12.8‰, and in two cases, winter δ^13^C values reach − 14‰ (TAÏ Ovis 10 and 40). At Gazel, 11 out of the 14 analyzed teeth also show winter δ^13^C values below − 12.8‰, with the lowest reaching − 13.6‰ (Table S2 and Figure S1–S3). These δ^13^C values fall within the threshold defined as potentially indicating a significant dietary contribution from forest resources.

## Discussion

### Autumnal lambing: sheep physiology and human agency

The occurrence of autumnal lambing in Early Neolithic contexts at Taï and Gazel constitutes the earliest evidence of this reproductive behavior in sheep in the Western Mediterranean region, where extended lambing periods and de-seasoning without artificial treatments are current practices today^8,11,17,18^. Autumnal births have been reported during the Chalcolithic period elsewhere in Europe but only as isolated cases: these are exceptions in assemblages where spring lambing appears to have been the general rule^12,53^. In the present investigation, where the limited sample size is nevertheless similar to previous studies, the relative proportion of autumnal lambing is high, suggesting a well-established practice.

Autumnal lambing demonstrates an extended breeding season for Early Neolithic sheep in Southern France, in comparison with present-day and Neolithic European sheep living in higher latitudes^12^^,^ but similar to present-day western Mediterranean breeds. Exactly how far back this physiological capability goes is a central question for understanding the reasons for its emergence, including heritage from the wild ancestor (*Ovis orientalis*), modifications driven by domestication due to selection and animal diet management, as well as the climatic framework. Consolidated data on birth seasonality in the early Holocene mouflon—the ancestor of domestic sheep – is currently lacking. Following the same analytical procedure as the one applied in the present study, stable isotope analyses of *Ovis orientalis* teeth at the Epigravettian hunting campsite of Kalavan (north-eastern Armenia) demonstrated a reduced birth period for mouflons around 12,000 BCE^61^. However, both the environmental framework (Late Glacial) and wild sheep ethology (vertical mobility) may have engendered specific pressure on their reproductive behavior. The lambing season of the present-day mouflon in the Near East (*Ovis orientalis gmelini*) is very short (1–2 months), and occurs in the spring^62^. Early domestic sheep at Tell Halula in the Middle Euphrates Valley, dated to ca 7,500 cal BCE (Late Pre-Pottery Neolithic B) were shown to have a similar short period of birth (2.5 months)^63^. By contrast, an extended lambing period was confirmed in the Pre-Pottery Neolithic of Cyprus (7,600–6,900 cal BCE), which included autumn/winter births^64^. Both contexts are located at a latitude below 35°N, where present-day sheep tend to have a short period of sexual rest^65^. It is interesting to note that in feral domestic sheep (i.e., the European mouflon) currently found in Cyprus, Corsica and Sardinia, births occur over a short time period in spring^66–68^. Experiments on European mouflon kept under natural photoperiod conditions in Madrid, Spain (40°N), involving the monitoring of hormonal profiles in females, demonstrated extended ovulatory activity, starting in October and terminating in the spring^69^. The apparent discrepancy between a prolonged fertility season and a short lambing period may be explained by the role of social interaction in regulating seasonal reproduction in the mouflon. In the wild, males and females are segregated when females are not sexually active, and female and male herds are reunited at the start of the mating season^70^. If mating occurs within a short time after reuniting, this would explain a restricted lambing season. This implies that autumnal lambing in domestic herds not only involves the physiological capacity for a prolonged fertility period, but also adequate management of interactions between females and males, most likely in the form of the separation of females and males, and reuniting them towards the end of the fertility period.

Assuming that the observed extended fertility season in Early Neolithic Western Mediterranean sheep differed from that of their wild ancestors, this physiological character would result from domestication, but the driving mechanisms—i.e., the role of environmental factors and human selection—remain difficult to determine. Although the photoperiod is generally accepted as the primary environmental cue influencing seasonal reproductive patterns in sheep, a genetic basis for photoresponsiveness has been argued for and demonstrated in both sheep and European mouflon^10,71–73^. Indeed, sheep breeds in the Mediterranean area show different responses to photoperiod cues^74^. For example, Manchega and Lacaune ewes appear to be much more sensitive to photoperiod cues than Ripollessa ewes or present-day European mouflon ewes raised in the same locations in Spain^18^. This induces a longer duration of seasonal ovulatory activity in breeds with higher sensitivity to changes in the photoperiod. Human-controlled genetic selection is assumed to contribute to the longer mating season and greater prolificacy in Mediterranean sheep, as well as other aspects of the breeds such as, docility, color, productive rates and milk^18,75,76^. On the other hand, genetic origins confer phenotypical reproductive capacity, while environmental conditions influence their expression^77^. Although the heritability of fertility traits is low in sheep^78,79^, the onset, offset and duration of the breeding season can still theoretically be selected^80^. The simplest way for herders to operate selection for autumnal lambing would have been to keep the autumn offspring. Although both young females and males could have been selected for this purpose, operating selection on the females would probably have been more intuitive to Neolithic herders to produce autumn lambing females. Even under these circumstances, the process would thus require many generations, but could still be envisaged on a long-term scale.

### The economic outcomes of autumnal lambing

A prolonged lambing period is preferred by modern sheep farmers in order to extend the availability of sheep products throughout the year^10,13^. Early Neolithic farmers who settled in Southern France could also have pursued these economically attractive practices. These seasonal bi-modulated reproductive patterns might have brought important advantages, enabling the group to balance the timing of production and consumption throughout the year, and may also have reduced risks for the survival of the herd by scheduling the harvesting of animal resources throughout a longer period in the year. Newborn lambs and dairy products with high fat and animal protein content could be planned on a longer time scale, even though this probably resulted in a lower production rate for lactation during late autumn and winter^80^. Nevertheless, at the Taï and Gazel sites, the caprines mortality profiles suggest that both milk and meat could have been exploited by Early Neolithic groups (Bréhard et al. in press).

Regarding herd demographic dynamics, modern studies have demonstrated that autumn lambs reach puberty later than lambs born in the spring, which is the period during which plant resources are optimal^69,81–83^. In consequence, autumnal lambing delays the incorporation of new sexually mature individuals into the herd. Today, the spring lamb reaches puberty during its first breeding period, whereas in autumn lambs, the length of the prepubertal period is longer and they may not reach puberty before the second breeding season. However, in traditional husbandries, sheep are not mated until their second year, when they are 18 to 24 months old^84–86^, whereas they are mated during their first year in present-day industrial husbandry. Consequently, it is unlikely that this delay curbed herd growth rate in Neolithic husbandries.

### Winter foddering

The data presented here provide early evidence of sheep winter foddering with a significant contribution of resources from forested areas. At the time of the Early Neolithic occupation at Gazel, mixed deciduous oak and maple tree woods dominated the surrounding landscape^87,88^. Carpological remains include hazelnuts, acorns and grapes (L. Bouby, pers. comm.). The archaeobotanical record at Taï also attests to a non-homogeneous forest component, including open Mediterranean evergreen oak woods, but also a potentially denser alluvial forest near the Gardon River^36^. The archaeobotanical assemblage at the Taï site delivered a relatively high number of wild plant species showing great diversity^36^. The generally better state of preservation of these remains compared to those of cereals and pulses suggests that the wild plant assemblage did not follow the same formation process. Given that this assemblage also includes a fair number of species not considered to be edible for humans, the presence of wild plants at the site could be linked to animal husbandry. In particular, the seeds and whole fruits of the hazel tree, elder, juniper, pistachio tree and evergreen oak could have been brought to the site with leafy branches and given to animals as fodder or litter^36^.

In this context, it is appropriate to combine the isotopic ratios measured in the Taï sheep tooth enamel with the archaeobotanical evidence. First of all, the low δ^13^C values attest to the presence of a woodland component in the landscape, dense enough to create a “canopy effect”^60^. Secondly, the δ^13^C values suggest that sheep fed on resources from the forest, but cannot distinguish between herding under forest canopy and provisioning with culled fodder. Archaeobotany demonstrates that leafy branches were brought to the site. Therefore, these two indications suggest that these branches were actually used as fodder (but could still also have been used as litter). Lastly, the presence of fruit suggests that leafy branches were collected in late summer and possibly until early winter^36^^,^ while the stable isotope evidence demonstrates that fodder – or at least the forest component, was mostly fed to sheep during winter. This arboreal fodder would have necessarily been stored between collecting and provisioning to sheep.

**Figure 3:**
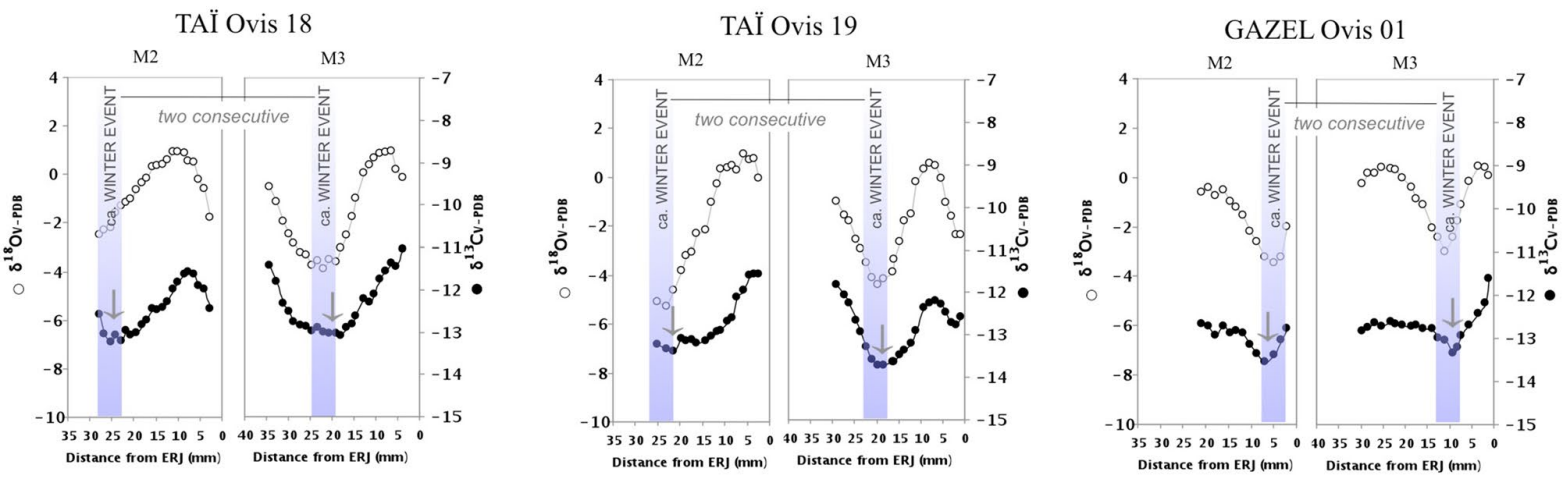
Sequential δ^13^C (black) and δ^18^O (white) values measured in enamel bioapatite of the second (M2) and third (M3) molars of three specimens (TAÏ Ovis 18 and TAÏ Ovis 19, GAZEL Ovis 01), showing variations in phase with the δ^13^C and δ^18^O sequences. The low δ^13^C values occurring at the same time of the minimum δ^18^O peak events indicate winter foddering during two consecutive years.

The cutting, transport and storage of leafy fodder would have required labor and specific organization, which may potentially have overlapped with other tasks, including the summer harvest or the autumn sowing of winter cereals (as shown at the Taï site^36^. It is difficult to evaluate just how much labor was invested as this would primarily have been a matter of scale. The number of sheep, the length of the foddering period, the proportion of leafy fodder in the sheep diet and the distance at which fodder was harvested, are key parameters^25^^,^ none of which can be accurately determined. Forest resources were fed to sheep in significant quantities and over a sufficiently long period to affect the stable isotope record. Additionally, at both sites, winter foddering appears to be a recurrent seasonal practice. On an individual scale, it is mirrored in both sampled teeth (M2 + M3), providing records for two consecutive winters (Fig. 3). On the scale of the sampled population, the practice is reflected in several, not necessarily contemporaneous specimens over the time span when the sites were settled. Overall, this would suggest a more systematic and intensive practice rather than opportunistic activity during brief periods of severe winters. These conclusions echo previous findings in other Neolithic contexts in Southern France where the archaeobotanical evidence attests to tree leaf supplies to stalled domestic herds^89–91^. In terms of the lambing pattern at Taï and Gazel, providing additional fodder to sheep over winter would have helped maintain good body weight throughout the winter, promoting good conditions for spring mating leading to autumnal lambing. At the same time, it would have been beneficial to ewes in the last period of gestation for late winter/early spring lambing.

## Conclusion

The results of our study are threefold. Firstly (i), they relate to animal physiology, as they demonstrate the capacity of Early Neolithic sheep from Southern France to give birth in autumn, at a time of year currently considered to be “off-season” *i.e.,* contrasting sharply with the general pattern of spring lambing at higher latitudes in Europe. This would constitute the earliest evidence for the genetic selection of specimens with prolonged reproductive fertility^18^^,^ which may therefore have been practiced since the earliest presence of sheep in the Western Mediterranean, at the least. These ancient systems may or may not eventually have led to the origins of present-day Mediterranean breeds with specific photoresponsive aptitudes. Although regional particularisms in the Mediterranean area, due to geographic compartmentalization, and the history of human migrations and trade, have favored the consistency of genetic specificity in local animal breeds^7^^,^ it is nonetheless beyond the scope of this study to establish a direct link between present-day and Early Neolithic sheep in Southern France. Secondly (ii), these results relate to zootechnical knowledge: autumnal lambing is the outcome of the intentional management of female and male interactions within the herd, probably by means of separating both sexes and reuniting them towards the end of the fertility period. Managing sheep socio-sexual cues in this manner would have required good knowledge of the timing of the fertility cycle in ewes. Autumnal lambing may have been supported by the winter foddering of sheep, in order to maintain a good body weight for spring mating. Forest resources were used for this purpose, involving potentially important—although difficult to evaluate, task investment. Thirdly (iii), these results relate to paleoclimatology, as autumnal lambing would support the assumption that a Mediterranean type precipitation regime existed at this time^19^. Indeed, Early Neolithic ewes could not have given birth in the autumn without a secure availability of pasture resources at that time of the year in order to support lactation.

The autumn lambing observed at Taï and Gazel occurs in both cases alongside more classical lambing at the end of winter to early spring. The subsequent expansion of the sheep birthing period should be taken into account when establishing the farming calendar, as the period of births and initiation of lactation necessitate increased monitoring by herders. It is very unlikely that females would have lambed twice a year. Rather, a system involving two groups of females giving birth at different times of the year should be envisaged, with sufficiently long periods of post-partum sexual rest to allow females to move from one group to the other, in order to enhance flexibility. Such a system would also enable empty ewes to lamb. In return for this investment, herders benefited from the distribution of seasonal animal resources over a longer period in the year, even though milk exploitation does not seem to have been substantial enough to induce specific demographic management. Caprine mortality profiles at the Taï and Gazel settlements suggest mixed exploitation for both meat and milk. In conclusion, the establishment of sheep husbandry systems involving autumnal lambing in the Early Neolithic of Southern France attests to a strong capacity to adapt to environmental contexts, which may have been a key to the successful expansion of agropastoral socio-economies across the wide range of climatic and ecological zones^92^ of the vast European continent. The specificities described in this study in terms of demographic and food management may also explain the multi-directional^5^ and arrhythmic^6,93^ character of the expansion of husbandry systems in the Western Mediterranean region. This scenario fits well with those proposed by Guilaine^94^^,^ who considered that these Early Neolithic communities successfully adapted to the advantages of new ecological niches in the Western Mediterranean.

## Materials and method

### Sampling, pre-treatment and IRMS analysis

The studied sample consists of nine sheep from the Taï site and 12 sheep from the Gazel site. One second (M2) or one third molar (M3), or both molars, were taken from each sheep, totalizing 28 teeth (Table S1). Sequential sampling of enamel along the tooth growth axis was conducted by drilling from the tooth surface^39^. As the timing of the formation of second and third molars is different, the signals in both teeth are complementary. Sampling combining the slightly worn M2 with the unworn but fully mineralized M3 molars from a given specimen provides a record of the first two years of the life of the specimen^53^^,^^95,96^.

Enamel powders were treated with 0.1 M acetic acid (0.1 ml solution/mg enamel) for 4 h at room temperature to eliminate diagenetic carbonates. Pretreated powders were analyzed individually on a Thermo Kiel IV device interfaced with a Thermo Delta V Advantage isotope ratio mass spectrometer. The δ^13^C and δ^18^O values are expressed versus the Vienna-Pee Dee Belemnite standard. They were corrected by comparison with a laboratory standard (*Marbre LM*), normalized in keeping with the NBS 19 international standard. Over the period of analyses, 192 *Marbre LM* samples gave a mean δ^13^C value of + 2.13 ± 0.01 ‰ (1σ) (expected value + 2.13 ‰) and a mean δ^18^O value of − 1.61 ± 0.03 ‰ (1σ) (expected value − 1.83 ‰). The mean analytical precision within each run was calculated from 6 to 8 measurements of the *Marbre LM* in each analytical series averaging 0.001 ± 0.011 ‰ for δ^13^C values and − 0.025 ± 0.025 ‰ for δ^18^O values.

### Modeling of δ^18^O sequences

In order to quantify inter-individual variability in the positioning of the maximum δ^18^O value in the tooth crown, the sequences of δ^18^O values were modeled using an equation derived from a cosine function^51^:

$$\delta^{{{18}}} {\text{O}}_{{\text{m}}} = {\text{ A}} \cdot {\cos}\left( {{2}\prod \cdot \left( {{\text{x}} - {\text{x}}_{0} } \right)/{\text{X}}} \right) + {\text{M}}$$

where δ^18^**O**_**m**_ is the modeled δ^18^O; ***x*** is the distance from the enamel-root junction; **X** is the period (in mm), or the length of tooth crown potentially formed over a whole annual cycle; **A** is the amplitude (= max–min/2) (in ‰); **x**_**0**_ is the delay (mm) ; δ^18^O attains maximum value when *x* = x_**0**_; **M** is the mean (= (max + min)/2) expressed in ‰.

The best match of the measured data with the parameters of the model was determined using an iterative method, and a minimization of the sum of the square of the difference between the model and the measurements (the method of least squares). Calculations were carried out using Microsoft Excel software.

The tooth size factor is eliminated through normalization of distances using the period **X** of the δ^18^O cycle. The position of the maximum δ^18^O values in the tooth crown is therefore expressed as **x**_**0**_**/X**. Inter-individual variability in (**x**_**0**_**/X)** reflects variability in the birth season^51^. Following Balasse et al. (in press), we use a circular representation of the data to reflect the cyclical nature of seasonality: January follows December, i.e. when (x_0_/X) reaches 1, it also reaches 0.

### Threshold δ^13^C values in sheep tooth enamel

The typical enamel δ^13^C value reflecting feeding on plants from open settings was determined using a mean δ^13^C value of − 27‰ for modern C_3_ plants^97,98^, corrected by + 1.5 ‰ to account for the fossil fuel effect^99^^,^^100^^,^ and by applying an enamel-diet ^13^C-enrichment factor (e*) of + 14.1‰^101^. The effect of the consumption of forest tree leaves as winter fodder on enamel δ^13^C values was estimated as follows: in the modern dense deciduous forest of Dourdan (France), δ^13^C values measured in roe deer bone collagen, reflecting δ^13^C values for ingested plants over a life span, average − 24.7‰^102^. An average value of − 29.7‰ may be estimated for plants^103^^,^ approximately 3‰ lower than the average values of − 27‰ expected for C_3_ plants in open areas. When corrected for the fossil fuel effect, this would lead to a value of − 28.2‰ in pre-industrial plants, or − 14.5‰ in the enamel of animals feeding on them. Therefore, significantly different δ^13^C values to the average value of − 11.8‰ expected for feeding in open settings, lowered to − 12.8‰ in winter (to account for a seasonal variation of approximately ± 1‰^58^^,^ closer to − 14.5‰, could therefore reflect a contribution of plant resources gathered in the forest.

SI “Materials and Methods” contains a more detailed description of the archaeological sites, osteological analyses and stable isotope methods.

## Supplementary information

Supplementary information
