# Supplementary material for: Early evidence of sheep lambing de-seasoning in the Western Mediterranean in the sixth millennium BCE
Source: Sci Rep. 2020 Jul 30;10:12798. doi: 10.1038/s41598-020-69576-w (PMC7393119; doi:10.1038/s41598-020-69576-w)
Supplement: Supplementary file 1 — Supplementary information [file 41598_2020_69576_MOESM1_ESM.doc]

SUPPORTING INFORMATION (SI) FOR

Early evidence of sheep lambing de-seasoning in the Western Mediterranean in the sixth millennium BCE

**AUTHORS**

C. TORNERO^1*^; m. Balasse^1^; S. BRÉHARD^1^; I. CARRÈRE^2^; D. FIORILLO^1^; J. GUILAINE^3^; J.-D. VIGNE^1^; c. MANEN^2^

**SI Materials and Methods**

*- Extended information for the Taï and Grotte du Gazel sites*

The site of *Taï* (43°19’33N, 2°25’17”E) is located in the Gard department, in the *Occitanie* region of southern France. The site is located about 54 m.a.s.l at the bottom of a ravine with a steep slope in the Gardon valley, very close to the River Rhône.

This site has been discussed in the archaeological records since the early 20th century^1–3^ and has recently been the subject of intensive archaeological research by a French scientific team directed by Claire Manen^4^. This Archaeological investigation has documented a long stratigraphic sequence from the Early to Late Neolithic period.

The *Epicardial* phases date to 5270-4990 cal BCE. This is the best represented archaeological phase at the site, giving a particularly eloquent testimony concerning how the implementation and development of agrarian societies was initially achieved in the Languedoc region. During this period the settlement was intensively used, providing evidence of occupation from both inside and outside the cave. A permanent occupation of the cave is demonstrated by the presence of storage pits, evidence for agriculture close to the site, and the nature of the archaeobotanical assemblage suggest a permanent occupation and the presence of domestic animals at the site at least most of the year^4,5^.

The excavation of the Early Neolithic levels of the *Taï* site (both the cave and porch area) provided a large sample of well preserved faunal remains. The number of identified specimens, NISP, is 3100 for mammals. This faunal assemblage is characterised by a high proportion of Lagomorpha remains, which are food refuse, but if these are excluded then the faunal spectrum is dominated by domestic sheep, goats and cattle (91% of the NISP for this reduced group). The caprines, predominately sheep, are clearly the main domestic species (71% of the NISP for this reduced group). Suids and wild carnivores have also been identified (9% of the resultant NISP) but they do not appear to play an important role in the economic system of the site (Bréhard et al., in press).

The *Grotte Gazel* (43°56’25’’N, 4°33’52”E) is located in the Sallèles-Cabardès, Languedoc-Roussillon, south-east region of France at approximately 250 m.a.s.l. The cave has been excavated from the 1960’s until the early 1990’s under the direction of Prof. J. Guilaine. A total of six different occupation levels have been identified (Guilaine, 1970), dating from the Upper Palaeolithic to the Late Bronze Age. The *Epicardial* levels investigated here are documented in Phases II and III and dated to 5350 to 5200 cal BCE and 5200 to 5050 cal BCE, respectively. The zooarchaeological analyses conducted in Phases II and III^6,7^, show that the faunal spectra are greatly dominated by domestic caprines (circa 60% of the NISP), as in the *Taï* site. However, contrary to the *Taï site*, large game such as aurochs and cervids also contribute to the economic system found at *Grotte Gazel* (circa 10% of the NISP).

Another significant difference between the two sites are the relative proportions of suids to domestic cattle, the former being more important (11% and 18%, respectively, of the NISP) than the latter at *Gazel*. Considering the study by D. Geddès in the 1980’s^6^, the mortality profile obtained from caprines remains suggests a type of exploitation based on either being a meat source: a third having been killed before they reached 12 months or utilized for reproductive purposes, a further third died after reaching 4 years age, while milk production cannot be discarded^6^.

*- Selection of sheep remains for isotopic analysis*

Sheep mandibles were identified from the faunal assemblages recovered at the *Taï* and *Grotte Gazel* sites, employing osteological criteria^8–12^. Samples selected for isotopic analyses belong to nine different individual specimens by considering the laterality, morphological characters and wear stage patterns

*- Mechanical sampling procedures in archaeological sheep teeth*

In the *Taï* assemblage, a total of nine specimens from the Early Neolithic were selected for isotopic analyses (Table_S1). Only the specimens with complete hemi-mandible and well-preserved dentition were selected for analysis, although in two cases (Ovis 30 and Ovis 40) incomplete hemi-mandible with only one molar left have also been selected. Only high crowned teeth with completely formed molar crowns were selected in order to achieve the longest temporal sequence. In five of the individuals selected the analysis could be performed on both the M2 and M3 (TAÏ Ovis 11, Ovis 18, Ovis 19, Ovis 20 and Ovis 21). However in TAÏ Ovis 09 and 30 the analysis was performed only on the M2 molars. In TAÏ Ovis 10 and 40 analysis was performed only on the M3 molars.

In the case of the *Grotte Gazel* assemblage, a total of 12 specimens were selected for analysis (see Table_S1). The same sampling criteria that were utilised for the remains from *Taï* were also employed for the *Grotte Gazel* remains. In this case all the teeth came from complete hemi-mandibles, and all specimens had their M2 molars analysed, except specimens GAZEL Ovis 01 and 02 in which the M3 molar was analysed as well.

The sheep molar from both archaeological sites were prepared for isotopic analyses utilising the same processes. The molars were extracted from complete hemi-mandibles from a small incision in the lingual side of the corpus of the mandible. The molar’s enamel were sampled using a serial or sequential extraction perpendicular to the tooth’s growth axis, from the apex to the enamel root junction (ERJ) along the whole crown height. This was performed on the buccal side of the tooth, on the anterior (M2) or middle (M3) lobes. On the M2, number of samples drilled out range from 12 to 20 while for the M3 this range was from 18 to 24. This variability is explained by different crown heights induced by the different wear stages. The drilled enamel bands involved were less than one millimeter wide and the bands were distanced at intervals of approximately <1 to 1.5 mm. The total number of samples drilled was 518. The positions of samples along each tooth crown were noted as the distance (in mm) from the ERJ, and all of them related to the final development stage of the tooth crown.

*- Modern reference sets for Sheep*

All modern reference data sets used in this study were analysed following the same methodological approach as detailed above. These references have already been published elsewhere^13–16^. Briefly, we considered the information from the four different herds which were composed of *Pré-Alpes du Sud* sheep. They were all raised on an experimental farm in South-eastern France (Digne, *Alpes de Haute Provence*, France). The specimens sampled were lambed between January to early February of 2000 and 2001 (CAR 26 and 1216), and in mid-September of 2000 (CAR 522 and 562)^13,17^. These specimens gave normalised *x_0_*/*X* results of 0.12 to 0.14 for the late winter lambing’s and 0.75 to 0.76 for mid autumnal lambing’s.

The Rousand reference set (ROU) were composed of sheep from a Shetland cross. They were raised on a working farm on the island of Rousay in the Orkneys. The specimens sampled (n=9) were lambed between the end of April and beginning of May 2003. The normalised *x_0_*/*X* results gave a range from 0.19 to 0.33 for all specimens analysed^18^.

The Selgua reference set (XT) is composed of Xisqueta sheep raised on a working farm in the Ebro Valley (Selgua, Zaragoza-Aragón; Spain). The two specimens sampled (XT 01 and 02) were born between the 1st and 16th October 2013^15^. The sequences of their δ^18^O values were modelled and their normalised *x_0_*/*X* results are 0.83 and 0.88.

Finally, the Kemenez reference data set (KMZ) are composed of an Ouessant and Landes de Bretagne sheep cross. They were raised on the island of Kemenez,Molène, Finistère; France. The three specimens analysed were lambed in February and their normalised *x_0_*/*X* results were 0.19 to 0.20.

The normalised *x_0_*/*X* results from the different modern sheep herds show a good agreement with the course of the annual cycle and the known date of birth of specimens analysed^16,19^. The difference in molar development between the modern sampled sheep herds, is not to exclude the variability of the sample sets and that of normal molar development but is assumed to be of little significance in this study.

**SI references**

1. Mingaud, G., 1905. Au sujet d’objets préhistoriques découverts dans la grotte du Taï. Bulletin de la société d’étude des sciences naturelles de Nîmes, 1905, p. XXVII.

2. Pialat, A., 1907. La grotte du Taï dans la vallée inférieure du Gardon. L’Homme préhistorique, p. 364-368. in.

3. Coste A. and Gutherz, X., 1976. Découverte de la phase récente de la culture cardiale dans les garrigues de Nîmes (Gard), Bulletin de la Société Préhistorique Française, 73(8):246-250.

4. Manen C, Bouby L, Carrère I, Coularou J, Devillers B,Muller C, Perrin T, Sordoillet D, Vigne J-D, Voruz J-L. 2004. Nouvelles données sur le Néolithiqueancien gardois: résultats des campagnes de fouille 2001–2002 de la grotte du Taï (Remoulins). In: Dartevelle H, editor. Auvergne et Midi–Actualité de la recherche. Actes des cinquièmes Rencontres Méridionales de Préhistoire Récente, Clermont-Ferrand (Puy-de-Dôme), 8 et 9 novembre 2002. Cressensac: Préhistoire du Sud-Ouest. p. 321–6. in.

5. Bouby, L.; Durand, F.; Rousselet, O.; Manen, C. 2019. Early farming economy in Mediterranean France: fruit and seed remains from the Early to Late Neolithic levels of the site of Taï (ca 5300-3500 cal BC)., Vegetation History and Archaeobotany 28:(1):17–34.

6. *Geddès, D. 1980. De la chasse au troupeau en Méditerranée Occidentale: les débuts del’élevage dans le basin de l’Aude. Toulouse, École des Hautes Etudes en Sciences Sociales, France.*

7. Rowley-Conwy P., Gourichon L., Helmer D. & Vigne J.-D., 2013. Early domestic animals in Italy, Istria, the Tyrrhenian islands and Southern France. In: S. College, J. Conolly, K. Dobney, K. Manning & S. Shennan eds. The Origins and Spread of Domestic Animals in Southwest Asia and Europe. Left Coast Press, Walnut Creek, California, p. 161-194. in.

8. Helmer D., 2000. Discrimination des genres Ovis et Capra à l’aide des prémolaires inférieures 3 et 4 et interprétation des âges d’abattages: l’exemple de Dikili Tash (Grèce), Anthropozoologica 31, 29-38.

9. Halstead, P., 1998. Ask the fellows who lop the hay: leaf-fodder in the mountains of northwest Greece. Rural History, 9 (2): 211-234.

10. Balasse, M., Ambrose, S. H., Smith, A. B., Price, T.D., 2002. The seasonal mobility model for prehistoric herders in the south-western Cape of South Africa assessed by isotopic analysis of sheep tooth enamel. Journal of Archaeological Science 29(9), 917-932.

11. Zeder, M. A. & Pilaar, S. E. Assessing the reliability of criteria used to identify mandibles and mandibular teeth in sheep, Ovis, and goats, Capra. *Journal of Archaeological Science* **37**, 225–242 (2010).

12. Gillis, R., Chaix, L. & Vigne, J.-D. An assessment of morphological criteria for discriminating sheep and goat mandibles on a large prehistoric archaeological assemblage (Kerma, Sudan). *Journal of Archaeological Science* **38**, 2324–2339 (2011).

13. Blaise, E. & Balasse, M. Seasonality and season of birth of modern and late Neolithic sheep from south-eastern France using tooth enamel δ18O analysis. *Journal of Archaeological Science* **38**, 3085–3093 (2011).

14. Balasse, M., Obein, G., Ughetto-Monfrin, J., Mainland, I. 2012a, Investigating seasonality and season of birth in past herds: a reference set of sheep enamel stable oxygen isotope ratios. Archaeometry 54, 349–368.

15. Tornero, C. *et al.* Vertical sheep mobility along the altitudinal gradient through stable isotope analyses in tooth molar bioapatite, meteoric water and pastures: A reference from the Ebro valley to the Central Pyrenees. *Quaternary International* **484**, 94–106 (2018).

16. Balasse, M., Tresset, A., Bălăşescu, A., Blaise, E., Tornero, C., Gandois, H., Fiorillo, D., Nyerges, É.Á., Frémondeau, D., Banffy, E., Ivanova, M., 2017, Animal Board Invited Review: Sheep birth distribution in past herds: a review for prehistoric Europe (6th to 3rd millennia BC), Animal 11 (12), 2229-2236.

17. *Blaise, E., 2010, Economie animale et gestion des troupeaux au Né olithique final en Provence : approche archéozoologique et contribution des analyses isotopiques de l’émail dentaire, Oxford : John & Erica Hedges Ltd., XVI+399 p. (British Archaeological Reports, International series 2080.*

18. Balasse, M., Obein, G., Ughetto-Monfrin, J. & Mainland, I. Investigating seasonality and season of birth in past herds: A reference set of sheep enamel stable oxygen isotope ratios. *Archaeometry* **54**, 349–368 (2012).

19. Tornero, C., Bǎlǎşescu, A., Ughetto-Monfrin, J., Voinea, V. & Balasse, M. Seasonality and season of birth in early Eneolithic sheep from Cheia (Romania): Methodological advances and implications for animal economy. *Journal of Archaeological Science* (2013) doi:10.1016/j.jas.2013.05.013.

[Table_S1]: *Specimens selected for this study. Site, archaeological sector, SU (archaeological code) and archaeological level. Wear Stage code is following Payne (1973).*

[Table_S2]: *Descriptive statistics for the results from stable isotope analysis of sheep enamel at Taï and Gazel. Lower second (M2) and third molars (M3). Mean, Maximum, Minimun and range of stable carbon (δ^13^C) and oxygen (δ^18^O) isotope ratios.*

[Table_S3]: *Results from the calculation of the best fit for combined variation of X (period), A (amplitude), x_0_ (delay) and M (mean), when the model is applied to δ^18^O values of sheep M2 teeth from Taï and Grotte Gazel. The Pearson’s correlation coefficient (R) between measured and modelled datasets.*


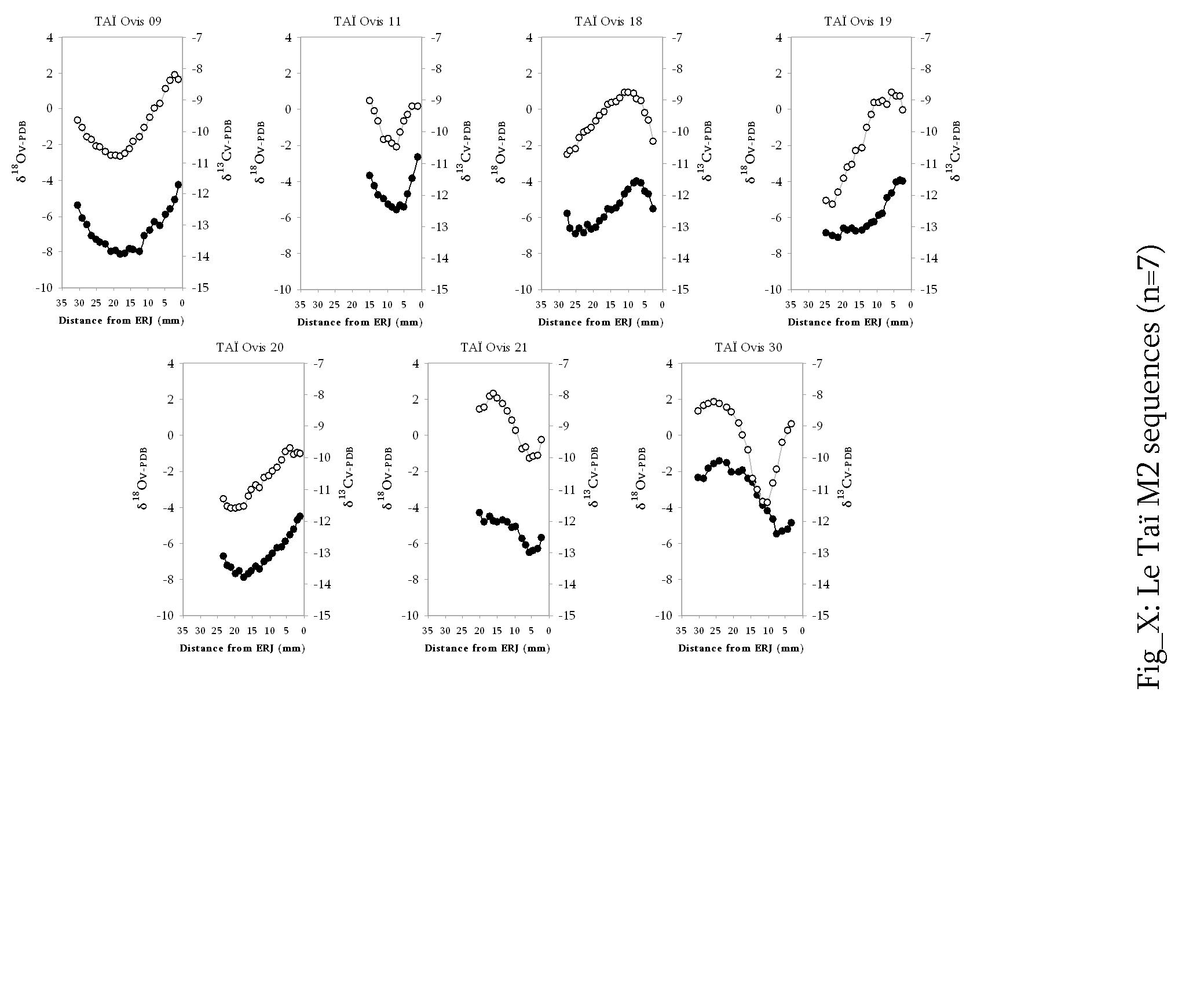


[Figure_S1]: *Sequential δ^13^C (black) and δ^18^O (white) values measured in enamel bioapatite of sheep second molars (M2) from Taï site.*


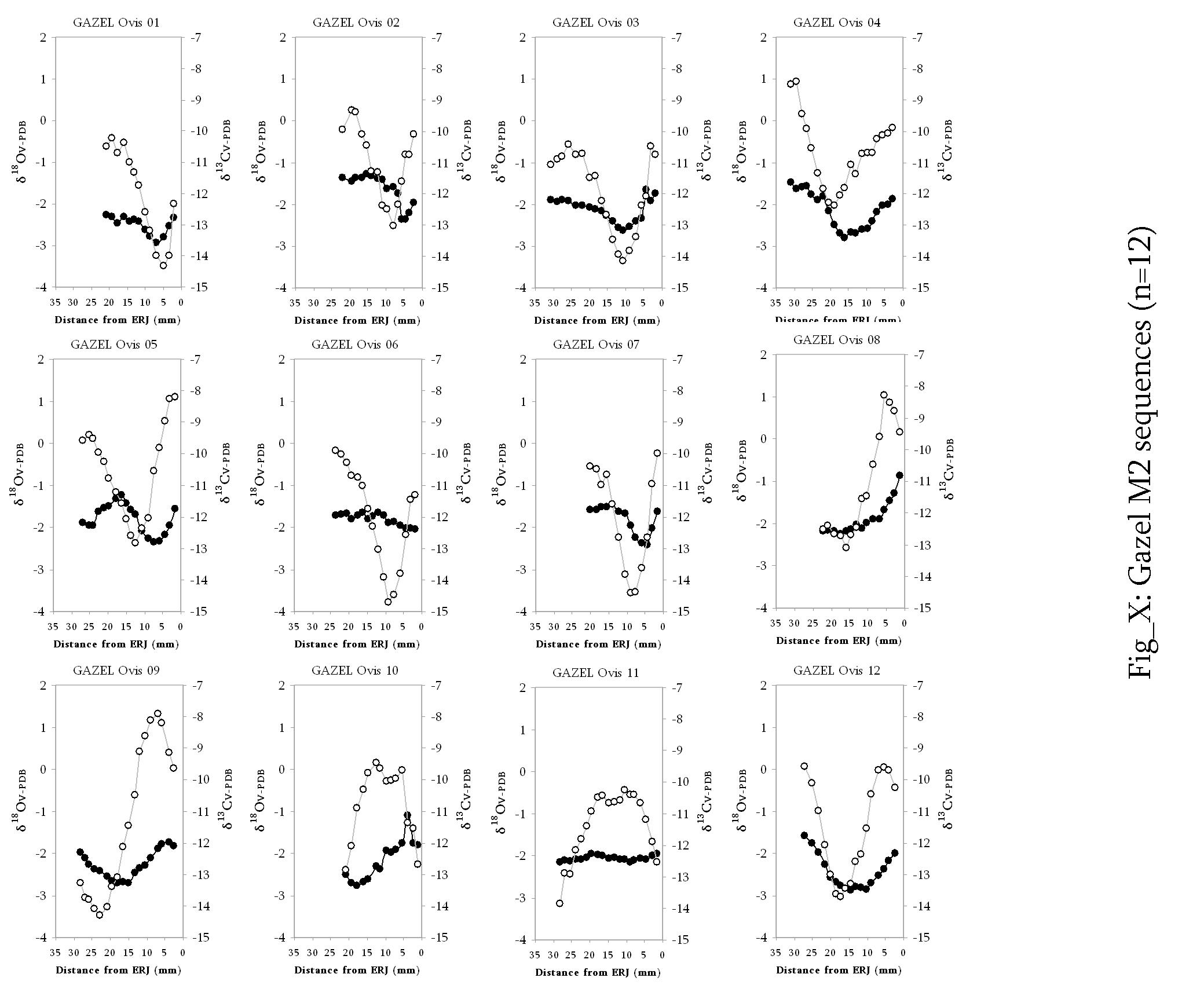


[Figure_S2]: *Sequential δ^13^C (black) and δ^18^O (white) values measured in enamel bioapatite of sheep second molars (M2) from Grotte Gazel site.*


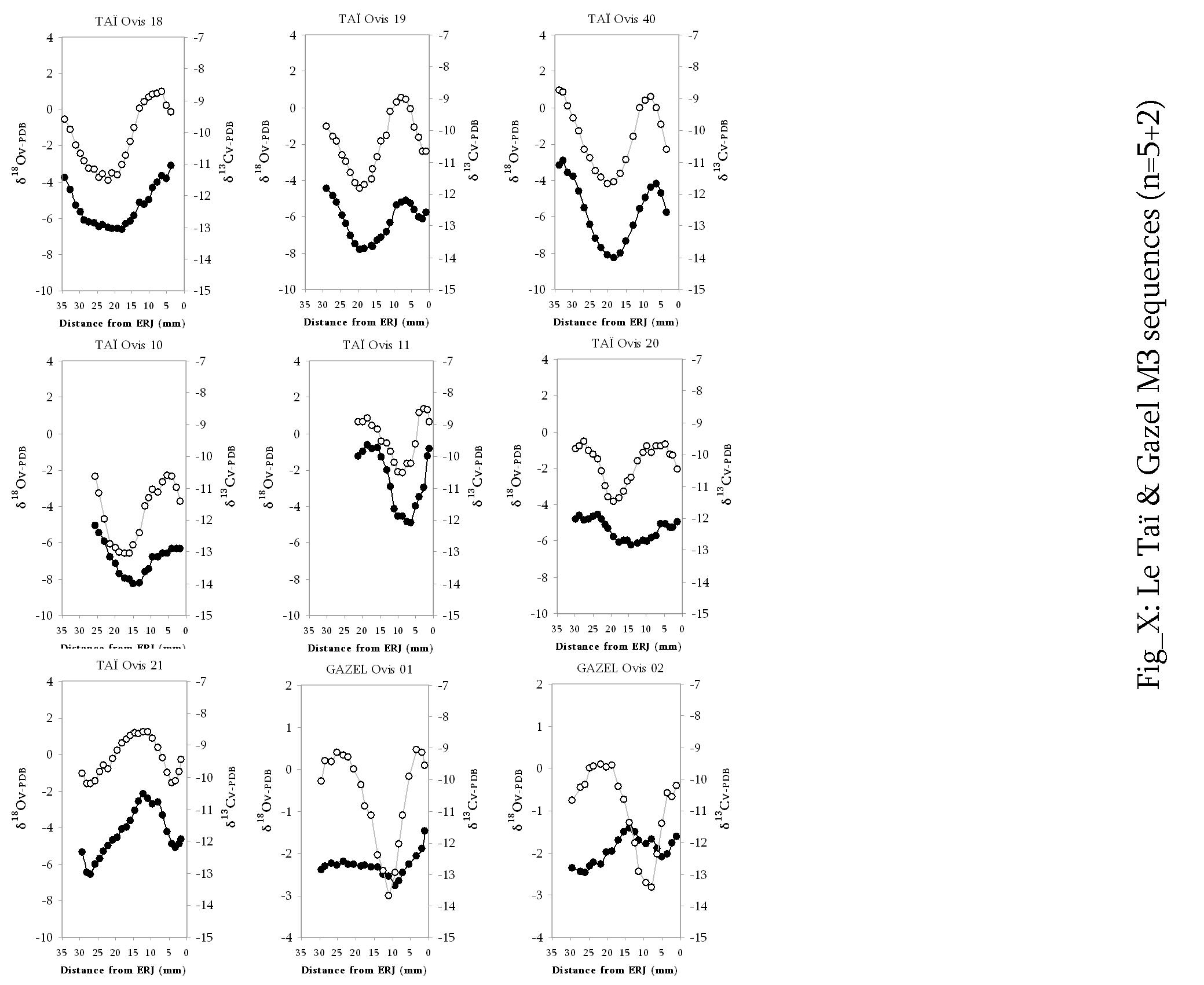


[Figure_S3]: *Sequential δ^13^C (black) and δ^18^O (white) values measured in enamel bioapatite of sheep third molars (M3) from Taï and Grotte Gazel sites.*
